# Evaluating post-traumatic growth among healthcare workers

**DOI:** 10.3934/publichealth.2025013

**Published:** 2025-02-10

**Authors:** Basant K. Puri, Anastasia Potoglou, Argyroula Kalaitzaki, Vasiliki Yotsidi, Maria Theodoratou

**Affiliations:** 1 Department of Molecular Biology, Cambridge Advanced Research, Cambridge, UK; 2 Department of Psychology, School of Health Sciences, Neapolis University Pafos, Pafos, Cyprus; 3 School of Social Sciences, Hellenic Open University, Patras, Greece; 4 Preliminary Health Care, Health Center of Lagadas, Thessaloniki Greece; 5 Social Work Department; Laboratory of Interdisciplinary Approaches for the Enhancement of Quality of Life; University Research Centre ‘Institute of AgriFood and Life Sciences', Hellenic Mediterranean University, Heraklion, Crete, Greece.; 6 Department of Psychology, School of Social Sciences, Panteion University of Social and Political Sciences, Athens, Greece

**Keywords:** post-traumatic growth, healthcare workers, PTGI, PTGI short-form, factor analysis

## Abstract

**Background:**

Recent studies have considered the psychological resilience and growth experienced by healthcare professionals, particularly those facing stressors and traumatic events.

**Aims:**

To study post-traumatic growth in healthcare workers caring for patients, determine the internal consistency of the Post-traumatic Growth Inventory (PTGI) and PTGI-Short Form (PTGI-SF) instruments, and carry out confirmatory analyses of their five-factor structures.

**Setting:**

Healthcare workers based in Greece.

**Methods:**

Cross-sectional design. Linear regression analysis with tested independent variables consisting of demographic, professional, health facility, and patient contact data. Confirmatory five-factor analyses of PTGI and PTGI-SF results were performed to validate their associated factors. Unidimensional reliability of the PTGI and PTGI-SF results was calculated.

**Results:**

The final regression model included sex and whether the internet was the source of health-related knowledge (*F*_2102_ = 11.979, *p* < 0.0001). The confirmatory factor analysis of the PTGI confirmed its five-factor structure (χ^2^_189_ = 1233.642, *p* < 0.0001), root mean square error of approximation (RMSEA = 0.229, *p* < 0.0001), and internal consistency (Cronbach *α* = 0.971). Similarly for the PTGI-SF (χ^2^_35_ = 535.965, *p* < 0.0001; RMSEA = 0.369, *p* < 0.0001; Cronbach *α* = 0.935).

**Conclusion:**

Being female and not using the internet as the principal source of information about diseases were each associated with increased post-traumatic growth. The internal consistencies of both the PTGI and the PTGI-SF were confirmed, as were the robustness of the five-factor structure of each instrument.

## 1. Background

Healthcare workers are frequently exposed to traumatic events and high-stress environments, which can significantly impact their mental health and well-being. Healthcare workers often witness distressing situations such as patient deaths, medical emergencies, and acts of violence within healthcare settings, leading to heightened levels of emotional distress and trauma symptoms [1]. Additionally, the demanding nature of their work, characterized by long hours, heavy workloads, and the pressure to make critical decisions under stress, contributes to chronic stress and burnout among healthcare workers [2]–[4]. Research has shown that prolonged exposure to these occupational stressors can increase the risk of psychological disorders, including post-traumatic stress disorder (PTSD), among healthcare professionals [2].

Moreover, healthcare workers may experience secondary trauma or vicarious traumatization as a result of empathetic engagement with patients' suffering and traumatic experiences. Studies have highlighted that healthcare professionals can develop symptoms of secondary trauma through repeated exposure to patients' traumatic narratives and emotional distress [3]. This phenomenon underscores the importance of recognizing the indirect impact of trauma on healthcare workers' mental health and implementing supportive interventions to mitigate its effects. By addressing these challenges, healthcare organizations can promote a culture of well-being and resilience among their staff, ultimately enhancing both patient care outcomes and staff retention rates [4].

The psychosocial effects of COVID-19, both during and after the pandemic, were widespread globally and among vulnerable groups, including healthcare workers. During the pandemic, healthcare workers faced increased work demands owing to high patient numbers, staff shortages, limited disease knowledge, lack of effective treatments, and heightened exposure risks [5],[6]. They were not only responsible for treating infected patients but also at risk of virus transmission and exposed to significant human suffering [7]. Witnessing such suffering qualifies as potentially traumatic, defined as exposure to actual or threatened death, serious injury, or sexual violence. Prolonged exposure to such events increases the risk of developing PTSD [8],[9]. Studies have reported elevated psychological symptoms such as stress, anxiety, depression, and PTSD among healthcare workers during the pandemic [10],[11]. These conditions, whether clinically diagnosed or subclinical, can profoundly impact overall functioning and the ability to work effectively in healthcare settings [12]–[14].

Stressful events, such as the recent pandemic, are associated with negative psychological effects on healthcare workers, including PTSD and burnout [14]–[24]. Notwithstanding the strong association between the experience of trauma and adverse psychological and physical sequelae, exposure to challenging traumatic events may be associated with positive changes in individuals, particularly in the domains of self-perception, interpersonal relationships, and one's philosophy of life [25].

In 1996, Tedeschi and Calhoun published the Posttraumatic Growth Inventory (PTGI) [26]. This is a 21-item inventory that quantifies the positive legacy of trauma, with each item being based on a six-point Likert response scored from zero (“I did not experience this change as a result of my crisis”) to five (“I experienced this change to a very great degree as a result of my crisis”) [26]. A principal components analysis carried out by Tedeschi and Calhoun yielded six factors with eigenvalues greater than unity, of which the following five were retained: factor I, relating to others; factor II, new possibilities; factor III, personal strength; factor IV, spiritual change; and factor V, appreciation of life [13]. The overall internal consistency of the PTGI was reported to be high (Cronbach *α* = 0.90), while that of individual factors varied from *α* = 0.67 for factor V to *α* = 0.85 for both factors I and IV [13]. Fourteen years later, Cann and colleagues (who included both Tedeschi and Calhoun) published a 10-item short form of the PTGI (PTGI-SF), which retained the five-factor structure of the original, with each factor consisting of two items from the full PTGI [27]. It should be noted, however, that other studies have not reached an agreement on the factor structure of PTGI, suggesting that there is/are one (e.g., Jozefiaková et al., 2022 [28]; Sheikh & Marotta, 2005 [29]), three (e.g., Taku et al., 2007 [30]), four (e.g., Dubuy et al., 2022 [31]), or five (e.g., Karanci et al., 2012 [32]; Lamela, et al., 2013 [33]; Prati & Pietrantoni, 2014 [34]; Teixeira & Pereira, 2013 [35]) factors.

Studies of post-traumatic growth of healthcare workers have recently been reviewed by Li and colleagues [36]. Following strict inclusion criteria, this review included a total of 36 papers from 12 countries and examined data mainly from the PTGI (including Hebrew, Turkish, and Chinese versions) and the PTGI-SF. In two included studies, the 25-item PTGI-X was used, which is a PTGI-derived inventory that accommodates cohorts for whom traditional religious beliefs, mapping to the spiritual change factor IV of the PTGI, are not so culturally important. The review reported moderate levels of post-traumatic growth in healthcare workers, with factor I having the highest dimension score.

The aims of this study were as follows: First, to study post-traumatic growth in healthcare workers caring for patients in the prefecture of Thessaloniki during the recent pandemic with respect to specific demographic variables. Second, to determine the internal consistency and carry out a confirmatory analysis of the five-factor structure of the PTGI results of this cohort of healthcare workers. Third, to check the internal consistency and carry out a confirmatory analysis of the five-factor structure of the PTGI-derived shortened form, the PTGI-SF, for the same cohort.

## 2. Methods

### 2.1. Study sample

A sample of 120 healthcare workers based in the prefecture of Thessaloniki (also known as Thessalonica and Saloniki), Macedonia, Greece, who were caring for patients, were invited to participate in this study. The study took place in 2022. The questionnaires were distributed using Google Forms. The questionnaires included questions regarding basic demographic information, the type and duration of professional service, the type of health facility constituting the place of employment, the level of contact with patients, the level of satisfaction with knowledge about diseases and stressful events, the source of knowledge about these issues, and the full 21-item PTGI (Greek version) [37].

The study followed the ethical standards and guidelines of the Research Ethics Committee of the Hellenic Open University, which granted ethical approval on 31/10/21, and of the Helsinki Declaration of 1964. Participation in the study was entirely voluntary. Participants gave full, informed consent. Anonymity of their personal data was guaranteed; participants were informed that data collected would only be used in an anonymized form for the purposes of research.

### 2.2. Statistical analyses

Each item of the PTGI, *Q_i_* (*i* = 1 to 21), was treated as a continuous variable. Thus, the PTGI response variable $\overset{21}{\underset{i=1}{\sum }}Q_{i}$ was continuous and assumed to have a linear relationship to predictor variables; predictor variable effects were assumed to be additive. The independence of residuals was checked by calculating the Durbin-Watson test statistic, *d*, to ascertain any evidence of autocorrelation. A Q-Q plot of standardized residuals against theoretical quantiles was employed to check that the residuals were normally distributed. A forward stepwise linear regression analysis was carried out with the PTGI total score as the dependent variable and the following independent variables: age, time in service in post, highest level of education, healthcare worker categories, number of children, exposure to COVID-19 patients, and what concerned the participants about COVID-19. A threshold of *p* = 0.05 was set for entry into the model. Confirmatory factor analysis (CFA) was performed to validate the factors associated with the PTGI and the PTGI-SF. Goodness-of-fit CFA indices included the chi-square test, the Bentler-Bonett normed fit index (NFI), the Bentler-Bonett non-normed fit index (NNFI), the comparative fit index (CFI), the root mean square error of approximation (RMSEA), and the standardized root mean square residual (SRMR). The range for the NFI, NNFI, and CFI was 0 to 1 in each case, with a value close to 1 representing a good fit for the model. RMSEA values between 0.05 and 0.08 also represent a good fit of the model. It was noted that the sample size was relatively very small for confirmatory analysis; group comparisons were therefore generally non-parametric. In particular, the five-factor CFA was carried out by defining each factor as a vector, ***x****_j_*, using the equation ***X*** = ***MQ***, in which ***X*** = [***x***_1_ ... ***x***_5_]^*T*^, ***M*** is the matrix

$$\left[\begin{array}{ccccccccccccccccccccc} 0 & 0 & 0 & 0 & 0 & 1 & 0 & 1 & 1 & 0 & 0 & 0 & 0 & 0 & 1 & 1 & 0 & 0 & 0 & 1 & 1\\ 0 & 0 & 1 & 0 & 0 & 0 & 1 & 0 & 0 & 0 & 1 & 0 & 0 & 1 & 0 & 0 & 1 & 0 & 0 & 0 & 0\\ 0 & 0 & 0 & 1 & 0 & 0 & 0 & 0 & 0 & 1 & 0 & 1 & 0 & 0 & 0 & 0 & 0 & 0 & 1 & 0 & 0\\ 0 & 0 & 0 & 0 & 1 & 0 & 0 & 0 & 0 & 0 & 0 & 0 & 0 & 0 & 0 & 0 & 0 & 1 & 0 & 0 & 0\\ 1 & 1 & 0 & 0 & 0 & 0 & 0 & 0 & 0 & 0 & 0 & 0 & 1 & 0 & 0 & 0 & 0 & 0 & 0 & 0 & 0 \end{array}\right]$$ (1)

and ***Q*** = [*λ*_1_*Q*_1_ ... *λ*_21_*Q*_21_]^*T*^, in which *λ_i_* are the corresponding loadings. For the corresponding five-factor CFA of the PTGI-SF, ***M*** was

$$\left[\begin{array}{ccccccccccccccccccccc} 0 & 0 & 0 & 0 & 0 & 0 & 0 & 1 & 0 & 0 & 0 & 0 & 0 & 0 & 0 & 0 & 0 & 0 & 0 & 1 & 0\\ 0 & 0 & 0 & 0 & 0 & 0 & 0 & 0 & 0 & 0 & 1 & 0 & 0 & 0 & 0 & 0 & 0 & 0 & 0 & 0 & 0\\ 0 & 0 & 0 & 0 & 0 & 0 & 0 & 0 & 0 & 1 & 0 & 0 & 0 & 0 & 0 & 0 & 0 & 0 & 1 & 0 & 0\\ 0 & 0 & 0 & 0 & 1 & 0 & 0 & 0 & 0 & 0 & 0 & 0 & 0 & 0 & 0 & 0 & 0 & 1 & 0 & 0 & 0\\ 1 & 1 & 0 & 0 & 0 & 0 & 0 & 0 & 0 & 0 & 0 & 0 & 0 & 0 & 0 & 0 & 0 & 0 & 0 & 0 & 0 \end{array}\right]$$ (2)

Unidimensional reliability was determined by calculating the Cronbach *α* coefficient.

Statistical analyses and plots were carried out using R v. 4.2.3 with the packages lmtest (Testing Linear Regression Models), matrixStats [Functions that Apply to Rows and Columns of Matrices (and to Vectors)], ggplot2 (Create Elegant Data Visualisations Using the Grammar of Graphics), lavaan (Latent Variable Analysis) and stats, in addition to the IDE JASP 0.18.3.0 [38]–[43].

## 3. Results

A total of 105 healthcare workers (17 male and 88 female) took part in this study. They ranged in age from 19 to 67 years, with a mean (standard error) age of 45 (1.1) years. The mean age of the male participants was 43.0 (3.1) years, matched with that of 45.4 (1.2) years of the female participants (*t* = 0.817, *df* = 103, *p* = 0.416). Their specialties were as follows: 19 doctors, 65 nurses, 5 midwives, and 16 others. The types of health facilities where they worked were: 31 in a general hospital, 7 in a university hospital, 54 in a health center, and 13 in a local health unit. The breakdown of the source of knowledge about diseases by sex is shown in Table 1.

**Table 1.** Source of knowledge about diseases; percentages for each sex and for the total values are given in parentheses.

Sex	Source of knowledge				
	Hospital	Scientific articles	Internet	Television	Other
Male (%)	7 (41)	8 (47)	2 (12)	0 (0)	0 (0)
Female (%)	37 (42)	19 (22)	22 (25)	5 (6)	5 (6)
Total (%)	44 (42)	27 (26)	24 (23)	5 (5)	5 (5)

**Figure 1.**
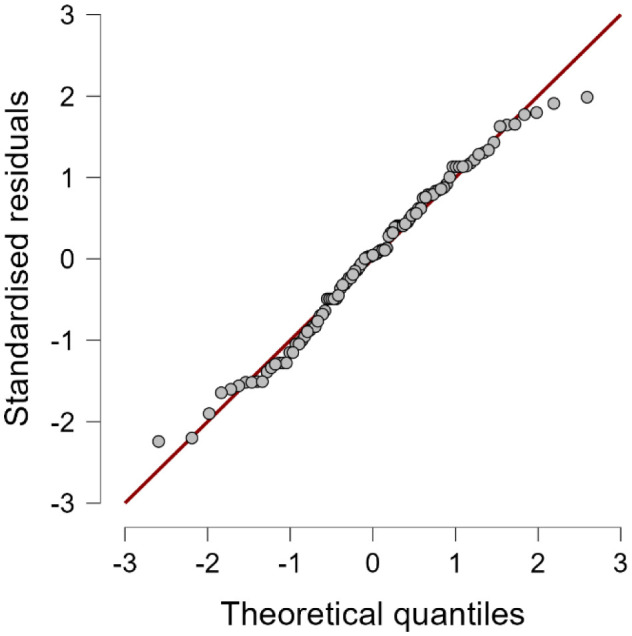
Q-Q plot.

The only variables that survived the stepwise linear regression analysis for inclusion in the final model were sex and whether the internet was the source of knowledge for health-related issues. The model was highly significant (*F* = 11.979, *df* = 2102; *p* < 0.0001) and is shown in Table 2. The value of *d*, 1.940, was non-significant (*p* = 0.740), while the Q-Q plot is shown in Figure 1.

**Table 2.** Linear regression model coefficients (and intercept) based on a normal (Gaussian) distribution, with the identity canonical link function and using the *t*-test to calculate the statistical significance of each coefficient (and the intercept). SE is the standard error of the mean.

Project	Coefficient	SE	*t*	*p*
Intercept	35.303	5.771	6.117	<0.0001
Female sex	18.204	6.306	2.887	0.005
Internet as the knowledge source	−23.573	5.532	−4.261	<0.0001

**Figure 2.**
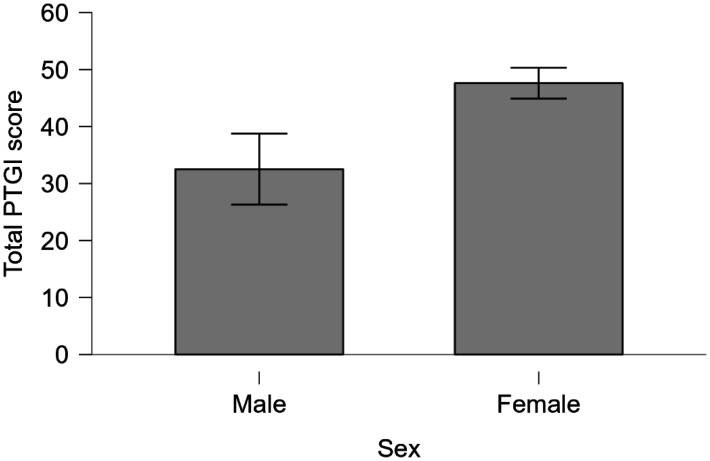
Sex difference in total PTGI scores. Bars indicate standard errors.

Post-hoc testing with the Mann-Whitney test showed that the mean total PTGI score in female healthcare workers, 47.614 (2.719), was significantly higher than that of 32.529 (6.229) in males (*U* = 503, *p* = 0.033) (Figure 2).

**Figure 3.**
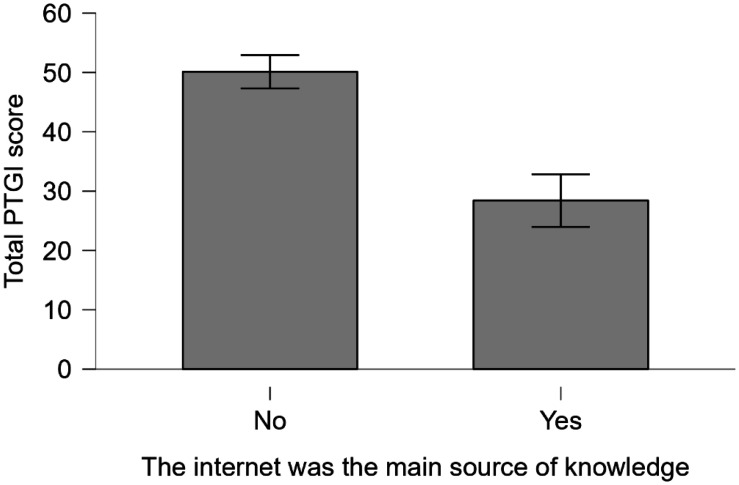
Total PTGI scores according to whether the internet was the main source of information regarding health-related issues. Bars indicate standard errors.

Similarly, and again using the Mann-Whitney test, the mean total PTGI score in those healthcare workers for whom the internet was the principal source of information about the coronavirus, 28.417 (4.432), was significantly lower than that of 50.136 (2.801) in those for whom it was not (*U* = 1453, *p* = 2.446 × 10^−4^) (Figure 3). There was no significant interaction between this variable and sex (*χ*^2^ = 1.415, *df* = 1, *p* = 0.234). The overall and grouped mean PTGI data are summarized in Table 3.

**Table 3.** PTGI scores, overall and grouped by sex, source of knowledge about the SARS-CoV2 coronavirus, and factor.

Project	*n*	*Mean*	*Standard error*
Overall	105	45.171	2.539
Sex			
Male	17	32.529	6.229
Female	88	47.614	2.719
Source of coronavirus knowledge			
Hospital	44	52.205	3.276
Scientific articles	27	47.593	5.890
Internet	24	28.417	4.432
Television	5	52.800	14.154
Other	5	43.000	8.689
Factor			
I	105	17.946	11.312
II	105	11.712	7.859
III	105	11.714	0.666
IV	105	6.894	0.473
V	105	9.342	0.528

The goodness-of-fit results for the CFA of the PTGI are shown in Table 4. The factor model was highly significant (*χ*^2^ = 1233.642, *df* = 189, *p* < 0.0001). The RMSEA measure of fit was 0.229 (90% confidence interval 0.217 to 0.242) and was highly significant (*p* < 0.0001).

**Table 4.** Goodness-of-fit results for the CFA of the PTGI.

Index	Value
χ^2^ (*df* = 189)	1233.642
NFI	0.520
NNFI	0.508
CFI	0.557
RMSEA	0.229
SRMSER	0.502

The CFA of the PTGI yielded the factor loadings shown in Table 5. All items for factors I, II, III, and V were highly significant (*p* < 0.0001). The PTGI scores by factor are given in Table 3.

The product-moment correlations between the factors are shown in Table 6. The Cronbach *α* was 0.971 (95% confidence interval 0.962–0.978) for the PTGI. The corresponding values for each of the factors are also given in Table 6.

The CFA of the PTGI-SF yielded the factor loadings shown in Table 7. Whilst none of the items was significant, overall, the factor model was highly significant (*χ*^2^ = 535.965, *df* = 35, *p* < 0.0001). The RMSEA was 0.369 (90% confidence interval 0.342–0.397) and was highly significant (*p* < 0.0001).

**Table 5.** Factor loadings (unstandardized) of the PTGI. Standard errors are given in parentheses.

Item	Factor I	Factor II	Factor III	Factor IV	Factor V
Q6	1.042 (0.129)				
Q8	1.162 (0.124)				
Q9	1.134 (0.132)				
Q15	1.410 (0.131)				
Q16	1.359 (0.118)				
Q20	1.314 (0.129)				
Q21	1.255 (0.123)				
Q3		1.035 (0.118)			
Q7		1.329 (0.130)			
Q14		1.307 (0.121)			
Q17		1.186 (0.134)			
Q11		1.209 (0.135)			
Q4			1.253 (0.127)		
Q10			1.284 (0.119)		
Q12			1.229 (0.118)		
Q19			1.306 (0.141)		
Q5				1.371	
Q18				1.625	
Q1					1.332 (0.136)
Q2					1.478 (0.125)
Q13					1.061 (0.135)

**Table 6.** Correlations between factors of the PTGI, with the reliability coefficients (Cronbach *α* values) given on the diagonal.

Factor	Factor I	Factor II	Factor III	Factor IV	Factor V
Factor I	0.930				
Factor II	0.911	0.896			
Factor III	0.849	0.856	0.899		
Factor IV	0.751	0.701	0.697	0.871	
Factor V	0.692	0.726	0.804	0.718	0.865

**Table 7.** Factor loadings of the PTGI-SF.

Item	Factor	Loading
Q8	I	1.210
Q20	I	1.228
Q11	II	1.316
Q7	II	1.293
Q10	III	1.220
Q19	III	1.290
Q5	IV	1.401
Q18	IV	1.590
Q1	V	1.427
Q2	V	1.380

The corresponding product-moment correlations between the factors of the PTGI-SF are shown in Table 8. The Cronbach *α* was 0.935 (95% confidence interval 0.914–0.952) for the PTGI-SF. The corresponding values for each of the factors are given in Table 8.

**Table 8.** Correlations between factors of the PTGI-SF, with the reliability coefficients (Cronbach *α* values) given on the diagonal.

Factor	Factor I	Factor II	Factor III	Factor IV	Factor V
Factor I	0.772				
Factor II	0.822	0.808			
Factor III	0.748	0.789	0.779		
Factor IV	0.659	0.628	0.666	0.871	
Factor V	0.522	0.618	0.680	0.656	0.886

## 4. Discussion

The psychosocial impacts of COVID-19 on healthcare workers have been profound and far-reaching. Their increased workloads owing to the high volume of infected patients, staff shortages, limited knowledge about the disease, lack of effective treatments, and high levels of exposure to the virus, combined with the responsibility of treating infected patients while being at risk of contracting the virus themselves, resulted in substantial psychological stress and exposure to traumatic events [1]–[4].

All three aims of this study have been fulfilled. First, it has been found that in the cohort of healthcare workers studied, being female was positively associated with post-traumatic growth, while using the internet as the principal source of COVID-19-related information had a negative association with post-traumatic growth. The sex difference finding is consistent with other similar findings, including the original sex difference in favor of women reported by Tedeschi and Calhoun at the time of the formulation of the PTGI [26],[27]. This suggests that female healthcare workers may have unique resilience factors or coping mechanisms that facilitate higher levels of post-traumatic growth compared with their male counterparts. This may be attributed to the divergences in coping mechanisms and social support systems that are more accessible or frequently utilized by women [44]. Women tend to have a higher propensity to seek out and benefit from social connections, which are crucial for fostering post-traumatic growth. Additionally, women often engage in more communal coping strategies [45]–[47], such as sharing experiences and emotions with others, which can enhance their ability to process traumatic events and derive meaning from them.

In terms of the findings related to internet health information-seeking, it seems reasonable to hypothesize that those for whom the internet was the principal source of information were more likely to search for online health-related information. Undoubtedly, utilizing online resources is essential for evidence-based practice [48],[49]. However, it would be interesting to carry out a more detailed study of the relationship between internet use and post-traumatic growth. Perhaps spending long periods accessing negative online information and opinions may negatively impact post-traumatic growth [49]. In breast cancer patients, for example, it has been reported that time online accessing cancer information is positively correlated with post-traumatic stress symptoms [50].

In terms of the second and third aims of the study, it is noteworthy that the five-factor structure of the PTGI was confirmed in the present cohort. The high internal consistency of the PTGI and all five factors point to the robustness of the original principal components analysis by Tedeschi and Calhoun [26]. The five-factor structure of the PTGI-SF was also confirmed. Again, there was a finding of high internal consistency for the PTGI-SF. The individual factors of the PTGI-SF generally had somewhat lower Cronbach *α* values, with wider confidence intervals, than the corresponding values for the full PTGI. This is not unexpected, given that each factor of the PTGI-SF only contains two items. Nevertheless, the present study again points to the robustness of the PTGI-SF.

This study has several important implications for understanding the post-traumatic growth of healthcare workers. First, it highlights the significant role of sex, suggesting that female healthcare workers may experience greater positive psychological changes following traumatic events. This finding aligns with previous research and underscores the need for approaches sensitive to a person's sex in supporting healthcare workers' mental health.

Second, the negative association between internet use as a principal source of COVID-19-related information and post-traumatic growth raises important questions about the impact of digital information consumption on mental health. While the internet is an invaluable tool for accessing health information, excessive or predominantly negative online content may contribute to increased stress and hinder post-traumatic growth [51]. Those who rely primarily on the internet for health information are more likely to encounter a substantial amount of negative information and opinions, which can exacerbate stress levels [52].

The potential adverse effects of prolonged exposure to negative online content should not be underestimated. A more detailed study of the relationship between internet use and post-traumatic growth would be beneficial. An excessive intake of negative health information online might impede the development of post-traumatic growth by perpetuating stress and anxiety. A deeper comprehension of this dynamic could facilitate the formulation of more efficacious guidelines for the consumption of online health information, thereby fostering healthier coping mechanisms and supporting positive psychological outcomes. Future research should explore this relationship further to develop strategies for promoting healthy digital information-seeking behaviors.

Lastly, the confirmation of the five-factor structure of the PTGI and the high internal consistency of the PTGI and PTGI-SF in this cohort affirm the robustness and reliability of these instruments in measuring post-traumatic growth among healthcare workers. These findings support the continued use of the PTGI and PTGI-SF in research and clinical practice to assess and promote post-traumatic growth in healthcare workers.

Important limitations of this study were the relatively small sample size, the ratio of females to males, and the small effect size.

## 5. Conclusion

In healthcare workers caring for patients, being female and not using the internet as the principal source of information about the COVID-19 coronavirus were both associated with increased post-traumatic growth. These findings underscore the importance of considering sex differences in the psychological response to traumatic events and suggest that reliance on digital information sources may have complex implications for mental health.

The study confirmed the internal consistencies of both the PTGI and the PTGI-SF in assessing post-traumatic growth among healthcare workers. The robustness of the five-factor structure of each instrument was also affirmed, supporting their continued use in both research and clinical settings to evaluate and promote positive psychological changes following trauma.

These insights are particularly relevant for developing targeted interventions aimed at enhancing resilience and post-traumatic growth in healthcare workers. Sex-specific strategies may be necessary to effectively support female healthcare workers, who appear to experience greater post-traumatic growth. Additionally, guiding healthcare workers toward healthy information-seeking behaviors and providing support in navigating online health information could mitigate the potential negative impact of excessive or negative digital content.

Future research should further investigate the dynamics of internet use and its relationship with post-traumatic growth to develop comprehensive strategies for mental health support. Understanding the nuanced effects of digital information consumption and the role of sex in post-traumatic growth will be crucial in preparing healthcare systems to support their staff in the face of ongoing and future challenges.

## Use of AI tools declaration

The authors declare they have not used Artificial Intelligence (AI) tools in the creation of this article.
